# 2,2′-(*p*-Phenyl­enedithio)diacetic acid

**DOI:** 10.1107/S1600536809015967

**Published:** 2009-05-07

**Authors:** Jian-Ling Yin, Yun-Long Feng

**Affiliations:** a Zhejiang Key Laboratory for Reactive Chemistry on Solid Surfaces, Institute of Physical Chemistry, Zhejiang Normal University, Jinhua, Zhejiang 321004, People’s Republic of China

## Abstract

The complete molecule of the title compound, C_10_H_10_O_4_S_2_, is generated by a crystallographic inversion centre. In the crystal, mol­ecules are linked into a one-dimensional chain by inter­molecular O—H⋯O hydrogen bonds.

## Related literature

For rigid aromatic carboxylic acids, see: Hu *et al.* (2006 ▶). The title compound, a new flexible aromatic multicarboxyl­ate acid, was designed and synthesized on the basis of the 1,4-benzene­bisoxyacetate (Li *et al.*, 2006 ▶) and phenyl­thio­acetate (Sandhu *et al.*, 1991 ▶) analogues.
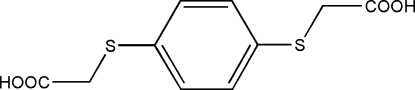


## Experimental

### 

#### Crystal data

C_10_H_10_O_4_S_2_
*M* *_r_* = 258.30
Triclinic, 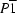

*a* = 5.5633 (4) Å
*b* = 6.9311 (5) Å
*c* = 7.6173 (6) Å
α = 79.809 (5)°
β = 70.738 (4)°
γ = 76.112 (4)°
*V* = 267.64 (3) Å^3^
*Z* = 1
Mo *K*α radiation
μ = 0.49 mm^−1^
*T* = 296 K
0.47 × 0.30 × 0.20 mm

#### Data collection

Bruker APEXII diffractometer
Absorption correction: multi-scan (*SADABS*; Sheldrick, 1996 ▶) *T*_min_ = 0.839, *T*_max_ = 0.908
3837 measured reflections
1209 independent reflections
1136 reflections with *I* > 2σ(*I*)
*R*_int_ = 0.016

#### Refinement

*R*[*F* ^2^ > 2σ(*F* ^2^)] = 0.027
*wR*(*F* ^2^) = 0.073
*S* = 1.09
1209 reflections
77 parameters
H atoms treated by a mixture of independent and constrained refinement
Δρ_max_ = 0.19 e Å^−3^
Δρ_min_ = −0.26 e Å^−3^

### 

Data collection: *APEX2* (Bruker, 2004 ▶); cell refinement: *SAINT* (Bruker, 2004 ▶); data reduction: *SAINT*; program(s) used to solve structure: *SHELXS97* (Sheldrick, 2008 ▶); program(s) used to refine structure: *SHELXL97* (Sheldrick, 2008 ▶); molecular graphics: *SHELXTL* (Sheldrick, 2008 ▶); software used to prepare material for publication: *SHELXL97*.

## Supplementary Material

Crystal structure: contains datablocks I, global. DOI: 10.1107/S1600536809015967/at2772sup1.cif

Structure factors: contains datablocks I. DOI: 10.1107/S1600536809015967/at2772Isup2.hkl

Additional supplementary materials:  crystallographic information; 3D view; checkCIF report

## Figures and Tables

**Table 1:** Hydrogen-bond geometry (Å, °)

*D*—H⋯*A*	*D*—H	H⋯*A*	*D*⋯*A*	*D*—H⋯*A*
O1—H1⋯O2^i^	0.82 (2)	1.82 (2)	2.6440 (14)	177 (2)

Symmetry code: (i) 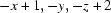.

**Fig. 1.**
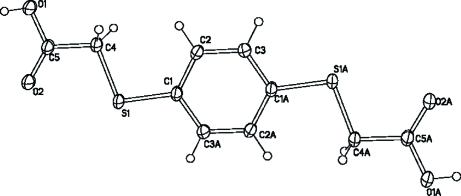
A view of the molecule of the compound (I), showing the atom-labelling scheme. displacement ellipsoids are shown at the 30% probability level.

**Fig. 2.**
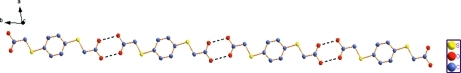
A view of the one-dimentional chain formed via O—H···O bydrogen bonds.

## supplementary crystallographic information

### Comment

Researches on the aromatic carboxylic acids mainly focused on the rigid acids (Hu *et al.*, 2006). Compared with the rigid acids, the flexible aromatic carboxylate acids contain more coordination sites and may lead to the versatile and novel metal-organic complexes. We successfully designed and synthesized a new flexible aromatic multicarboxylate acid,1,4-benzenebis(thioacetic acid) (I), on the basis of the 1,4-benzenebisoxyacetate (Li *et al.*, 2006) and phenylthioacetate (Sandhu *et al.*, 1991).

The compound (I) possesses two flexible carboxyl groups (Fig. 1). The centroid of the benzene ring of the molecule is an inversion centre and the asymmetric unit contains an half-molecule. The bond lengths and angles are as expected. In the crystal structure, intermolecular O—H···O hydrogen bonds link the molecules into a one-dimensional chain (Fig. 2).

### Experimental

The solution of 1,4-benzenebisthiol (7.11 g, 0.05 mol) in water (10 ml) neutralized with NaOH (4.00 g, 0.10 mol) was added to a 1:1 mixture of chloroacetic acid (18.90 g, 0.20 mol) and NaOH (8.00 g, 0.20 mol) with stirring to adjust the pH value of the mixture to *ca* 11 and refluxed at 363 K for 3 h. Then adjust the pH value to 2–3 with concentrated hydrochloric acid as soon as the reaction finished. The sample was filtrated, washed by water, then dried, the compound (I) was obtained with a yield of 80%.

### Refinement

The H atoms bonded to C atoms were positioned geometrically [aliphatic C—H = 0.97 Å and aromatic C—H = 0.93 Å, *U*_iso_(H) = 1.2*U*_eq_(C)]. The H atoms bonded to O atoms were located in a difference Fourier maps and refined with *U*_iso_(H) = 1.5*U*_eq_(O).

### Crystal data

**Table:**

C_10_H_10_O_4_S_2_	*Z* = 1
*M**_r_* = 258.30	*F*(000) = 134
Triclinic, *P*1	*D*_x_ = 1.603 Mg m^−^^3^
Hall symbol: -P 1	Mo *K*α radiation, λ = 0.71073 Å
*a* = 5.5633 (4) Å	Cell parameters from 2874 reflections
*b* = 6.9311 (5) Å	θ = 2.9–27.6°
*c* = 7.6173 (6) Å	µ = 0.49 mm^−^^1^
α = 79.809 (5)°	*T* = 296 K
β = 70.738 (4)°	Block, colourless
γ = 76.112 (4)°	0.47 × 0.30 × 0.20 mm
*V* = 267.64 (3) Å^3^	

### Data collection

**Table:**

Bruker APEXII diffractometer	1209 independent reflections
Radiation source: fine-focus sealed tube	1136 reflections with *I* > 2σ(*I*)
graphite	*R*_int_ = 0.016
ω scans	θ_max_ = 27.6°, θ_min_ = 2.9°
Absorption correction: multi-scan (*SADABS*; Sheldrick, 1996)	*h* = −7→7
*T*_min_ = 0.839, *T*_max_ = 0.908	*k* = −8→9
3837 measured reflections	*l* = −9→9

### Refinement

**Table:**

Refinement on *F*^2^	Primary atom site location: structure-invariant direct methods
Least-squares matrix: full	Secondary atom site location: difference Fourier map
*R*[*F*^2^ > 2σ(*F*^2^)] = 0.027	Hydrogen site location: inferred from neighbouring sites
*wR*(*F*^2^) = 0.073	H atoms treated by a mixture of independent and constrained refinement
*S* = 1.09	*w* = 1/[σ^2^(*F*_o_^2^) + (0.0362*P*)^2^ + 0.0845*P*] where *P* = (*F*_o_^2^ + 2*F*_c_^2^)/3
1209 reflections	(Δ/σ)_max_ < 0.001
77 parameters	Δρ_max_ = 0.19 e Å^−^^3^
0 restraints	Δρ_min_ = −0.26 e Å^−^^3^

### Special details

**Table:**

Geometry. All e.s.d.'s (except the e.s.d. in the dihedral angle between two l.s. planes) are estimated using the full covariance matrix. The cell e.s.d.'s are taken into account individually in the estimation of e.s.d.'s in distances, angles and torsion angles; correlations between e.s.d.'s in cell parameters are only used when they are defined by crystal symmetry. An approximate (isotropic) treatment of cell e.s.d.'s is used for estimating e.s.d.'s involving l.s. planes.
Refinement. Refinement of *F*^2^ against ALL reflections. The weighted *R*-factor *wR* and goodness of fit *S* are based on *F*^2^, conventional *R*-factors *R* are based on *F*, with *F* set to zero for negative *F*^2^. The threshold expression of *F*^2^ > σ(*F*^2^) is used only for calculating *R*-factors(gt) *etc*. and is not relevant to the choice of reflections for refinement. *R*-factors based on *F*^2^ are statistically about twice as large as those based on *F*, and *R*- factors based on ALL data will be even larger.

### Fractional atomic coordinates and isotropic or equivalent isotropic displacement parameters (Å^2^)

**Table:**

	*x*	*y*	*z*	*U*_iso_*/*U*_eq_	
S1	0.66238 (6)	0.55947 (5)	0.67832 (5)	0.03597 (14)	
O2	0.6441 (2)	0.17946 (15)	0.87948 (16)	0.0469 (3)	
O1	0.2166 (2)	0.19194 (15)	0.97977 (16)	0.0425 (3)	
H1	0.263 (4)	0.075 (4)	1.019 (3)	0.069 (7)*	
C1	0.5592 (3)	0.80504 (18)	0.58268 (18)	0.0294 (3)	
C2	0.3044 (3)	0.9090 (2)	0.6244 (2)	0.0396 (3)	
H2A	0.1718	0.8488	0.7077	0.048*	
C3	0.2472 (3)	1.1024 (2)	0.5422 (2)	0.0390 (3)	
H3A	0.0759	1.1712	0.5713	0.047*	
C4	0.3615 (3)	0.47918 (19)	0.80700 (19)	0.0327 (3)	
H4A	0.2621	0.5652	0.9046	0.039*	
H4B	0.2589	0.4866	0.7240	0.039*	
C5	0.4234 (3)	0.26763 (19)	0.89175 (19)	0.0327 (3)	

### Atomic displacement parameters (Å^2^)

**Table:**

	*U*^11^	*U*^22^	*U*^33^	*U*^12^	*U*^13^	*U*^23^
S1	0.0319 (2)	0.02282 (19)	0.0434 (2)	−0.00331 (13)	−0.00692 (15)	0.01037 (13)
O2	0.0351 (6)	0.0282 (5)	0.0625 (7)	−0.0045 (4)	−0.0070 (5)	0.0159 (5)
O1	0.0346 (5)	0.0263 (5)	0.0558 (7)	−0.0073 (4)	−0.0062 (5)	0.0111 (5)
C1	0.0329 (6)	0.0197 (6)	0.0314 (6)	−0.0045 (5)	−0.0078 (5)	0.0035 (5)
C2	0.0305 (7)	0.0276 (7)	0.0469 (8)	−0.0061 (5)	−0.0006 (6)	0.0106 (6)
C3	0.0278 (6)	0.0274 (7)	0.0494 (8)	−0.0018 (5)	−0.0038 (6)	0.0075 (6)
C4	0.0327 (7)	0.0227 (6)	0.0361 (7)	−0.0042 (5)	−0.0071 (5)	0.0060 (5)
C5	0.0351 (7)	0.0241 (6)	0.0337 (7)	−0.0061 (5)	−0.0065 (5)	0.0031 (5)

### Geometric parameters (Å, °)

**Table:**

S1—C1	1.7679 (12)	C2—C3	1.3860 (19)
S1—C4	1.8010 (14)	C2—H2A	0.9300
O2—C5	1.2139 (18)	C3—C1^i^	1.3843 (19)
O1—C5	1.3058 (17)	C3—H3A	0.9300
O1—H1	0.82 (2)	C4—C5	1.5015 (17)
C1—C3^i^	1.3843 (19)	C4—H4A	0.9700
C1—C2	1.3866 (19)	C4—H4B	0.9700
			
C1—S1—C4	103.14 (6)	C2—C3—H3A	119.4
C5—O1—H1	108.3 (16)	C5—C4—S1	108.38 (9)
C3^i^—C1—C2	118.87 (12)	C5—C4—H4A	110.0
C3^i^—C1—S1	115.93 (10)	S1—C4—H4A	110.0
C2—C1—S1	125.20 (10)	C5—C4—H4B	110.0
C3—C2—C1	120.01 (13)	S1—C4—H4B	110.0
C3—C2—H2A	120.0	H4A—C4—H4B	108.4
C1—C2—H2A	120.0	O2—C5—O1	124.48 (12)
C1^i^—C3—C2	121.12 (13)	O2—C5—C4	122.57 (12)
C1^i^—C3—H3A	119.4	O1—C5—C4	112.95 (12)
			
C4—S1—C1—C3^i^	−173.10 (11)	C1—C2—C3—C1^i^	0.2 (3)
C4—S1—C1—C2	7.47 (15)	C1—S1—C4—C5	178.43 (9)
C3^i^—C1—C2—C3	−0.2 (3)	S1—C4—C5—O2	4.17 (19)
S1—C1—C2—C3	179.18 (12)	S1—C4—C5—O1	−176.44 (10)

Symmetry codes: (i) −*x*+1, −*y*+2, −*z*+1.

### Hydrogen-bond geometry (Å, °)

**Table:**

*D*—H···*A*	*D*—H	H···*A*	*D*···*A*	*D*—H···*A*
O1—H1···O2^ii^	0.82 (2)	1.82 (2)	2.6440 (14)	177 (2)

Symmetry codes: (ii) −*x*+1, −*y*, −*z*+2.
